# Plasma Metabolites Forecast Occurrence and Prognosis for Patients With Diffuse Large B-Cell Lymphoma

**DOI:** 10.3389/fonc.2022.894891

**Published:** 2022-06-06

**Authors:** Fei Fei, Meihong Zheng, Zhenzhen Xu, Runbin Sun, Xin Chen, Bei Cao, Juan Li

**Affiliations:** ^1^ Phase I Clinical Trials Unit, Nanjing Drum Tower Hospital, The Affiliated Hospital of Nanjing University Medical School, Nanjing, China; ^2^ Department of General Surgery, Nanjing Drum Tower Hospital, The Affiliated Hospital of Nanjing University Medical School, Nanjing, China

**Keywords:** diffuse large B-cell lymphoma, metabolomics, metabolic heterogeneity, biomarker discovery, malate

## Abstract

**Background:**

Diffuse large B-cell lymphoma (DLBCL) is the most common non-Hodgkin’s lymphoma with considerable heterogeneity and different clinical prognosis. However, plasma metabomics used to forecast occurrence and prognosis of DLBCL are rarely addressed.

**Method:**

A total of 65 volunteers including 22 healthy controls (Ctrl), 25 DLBCL patients newly diagnosed (ND), and 18 DLBCL patients achieving complete remission (CR) were enrolled. A gas chromatography mass spectrometry-based untargeted plasma metabolomics analysis was performed.

**Results:**

Multivariate statistical analysis displayed distinct metabolic features among Crtl, ND, and CR groups. Surprisingly, metabolic profiles of newly diagnosed DLBCL patients undergoing different prognosis showed clear and distinctive clustering. Based on the candidate metabolic biomarkers (glucose and aspartate) and clinical indicators (lymphocyte, red blood count, and hemoglobin), a distinct diagnostic equation was established showing improved diagnostic performance with an area under curve of 0.936. The enrichment of citric acid cycle, deficiency of branched chain amino acid, methionine, and cysteine in newly diagnosed DLBCL patients was closely associated with poor prognosis. In addition, we found that malate and 2-hydroxy-2-methylbutyric acid were positively correlated with the baseline tumor metabolic parameters (metabolically active tumor volume and total lesion glycolysis), and the higher abundance of plasma malate, the poorer survival.

**Conclusion:**

Our preliminary data suggested plasma metabolomics study was informative to characterize the metabolic phenotypes and forecast occurrence and prognosis of DLBCL. Malate was identified as an unfavorable metabolic biomarker for prognosis-prediction of DLBCL, which provided a new insight on risk-stratification and therapeutic targets of DLBCL. More studies to confirm these associations and investigate potential mechanisms are in the process.

## Introduction

Diffuse large B-cell lymphoma (DLBCL), the most common non-Hodgkin’s lymphoma, displays great heterogeneity in the clinic (1). With the advent of anti-CD20-based immunochemotherapy, the survival of DLBCL patients has significantly improved. However, approximately 40% of patients with DLBCL still experience therapeutic failure resulting in progression or relapses (2). At present, the major risk-stratification for prognosis in the clinic is the clinically based International Prognostic Index (IPI), and the higher IPI scores, the worse prognosis (3). But the IPI system is less capable of identifying individual high-risk patients in the rituximab era (4). More recently, the molecular heterogeneity of DLBCLs is considered as a major factor influencing the response to therapy, like cell-of-origin classification and double-hit/triple-hit lymphomas (5, 6), while multiple studies failed to reproduce the predictive power (7–10). Hence, discovery of new therapeutic targets and prognostic markers is urgently needed to improve the current DLBCL-stratification system and guide the optimization of clinical therapy.

Reprogramming metabolism to ensure steady supply of intermediary metabolites for massive proliferation and malignant progression is a hallmark of a tumor (11). The exuberant metabolism of DLBCL cells was observed with increased uptake of glucose analog (^18^F-fluoro-2-deoxy-D-glucose, FDG), as well as up-regulated expression of glucose transporter and hexokinases (12, 13). Moreover, metabolic heterogeneity indicated by the distinct dependence on substrate in tumors is not only common in different types of tumors but also in subgroups within a tumor, which may influence therapeutic vulnerabilities and predict clinical outcomes (14, 15). There is increasing evidence that DLBCL is metabolically heterogeneous (16) and has been further classified into oxidative phosphorylation (OxPhos) cluster and B cell receptor (BCR) cluster (10). To date, very few studies have been reported to uncover distinct metabolic biomarkers involved in prognosis of DLBCL (17, 18). While due to the lack of healthy controls, more work is needed to verify reported results and discover new biomarkers. To our knowledge, plasma metabomics used to diagnose and prognosticate DLBCL are rarely addressed.

In this study, we profiled the plasma metabolome of peripheral plasma from DLBCL patients newly diagnosed, DLBCL patients achieving complete remission, and healthy controls to identify the potential metabolic markers for diagnosis. According to prognostic outcome after follow-up, we also systematically investigated the metabolic characteristics of two distinct groups subdivived from newly diagnosed patients. The prognostic biomarker and metabolic pathway correlated to clinical prognosis were further identified in DLBCL, which will throw light on metabolic pathogenesis and provide new alternative diagnoses and prognostic biomarkers for DLBCL.

## Materials and Methods

### Study Design

To ensure the accuracy of this experiment, volunteers with other malignancies and metabolic disease such as diabetes, and liver and kidney dysfunction were excluded. All DLBCL patients were diagnosed and reviewed by experienced hematopathologists and received an anti-CD20-based chemotherapy regimen. Finally, a total of 65 volunteers including 22 healthy controls (Ctrl), 25 DLBCL patients newly diagnosed (ND), and 18 DLBCL patients achieving complete remission (CR) were enrolled between Feburary 2018 and March 2019 in this analysis. This study was approved by the Medical Ethical Committee of Nanjing Drum Tower Hospital. Informed consent for plasma samples was obtained from all volunteers in accordance with the Helsinki Declaration.

Newly diagnosed DLBCL patients were further classified according to Han’s algorithm (the germinal center B cell subtype (GCB) vs. non-GCB) and the Ann Arbor stage (stage I-II vs. stage III-IV). Han’s algorithm was performed by senior pathologists based on immunohistochemistry (the expression of immune molecule markers including CD10, Bcl-6, and MUM1) (19). Efficacy of CR was evaluated based on the standards revised by the Lugano meeting (2014), including the length of target lesions (lymph nodes) was less than 1.5 cm, and no extranodal lesions were found; unmeasurable lesions disappeared; enlarged organs returned to normal; no new lesions; and bone marrow morphology was normal. Clinical characteristics of DLBCL patients and healthy volunteers involved are summarized in **Table 1**.

**Table 1:** The demographics and clinical characteristics of DLBCL patients and healthy volunteers.

Characteristics	Ctrl	ND	CR
Numbers	22	25	18
Age (years)	58.32 ± 14.70	59.68 ± 15.81	51.00 ± 16.48
Gender: M/F	13/9	12/13	9/9
**Classification**			
GCB/Non-GCB		13/12	
Stage I-II/III-IV		13/12	
**Blood routine**			
WBC (10^9^/L)	5.80 ± 1.13	5.98 ± 3.03	5.82 ± 1.84
Neutrophil (10^9^/L)	3.18 ± 0.82	4.10 ± 2.73	3.31 ± 1.12
Lymphocyte (10^9^/L)	2.07 ± 0.46	1.30 ± 0.57**	1.85 ± 0.80^#^
Monocyte (10^9^/L)	0.40 ± 0.12	0.41 ± 0.17	0.50 ± 0.16
RBC (10^12^/L)	4.75 ± 0.41	3.99 ± 0.61**	4.64 ± 0.63^##^
Hemoglobin (g/L)	142.32 ± 13.68	116.04 ± 18.37**	139.29 ± 17.11^##^
Platelet (10^9^/L)	218.50 ± 48.39	240.44 ± 94.83	198.88 ± 82.09
**Biochemistry analysis**			
Glutamic-pyruvic transaminase (U/L)	23.98 ± 18.13	21.37 ± 19.71	11.53 ± 7.54
Glutamic-oxalacetic transaminease (U/L)	21.41 ± 6.84	20.12 ± 7.96	14.08 ± 3.63
Alkaline phosphatase (U/L)	58.05 ± 11.96	97.32 ± 98.23	68.55 ± 18.16
Direct bilirubin (μmol/L)	2.57 ± 1.81	3.37 ± 2.75	2.20 ± 0.28
Total bilirubin (μmol/L)	10.06 ± 5.41	9.93 ± 5.42	8.98 ± 2.94
Lactate dehydrogenase (U/L)	197.05 ± 27.39	270.61 ± 241.19	195.50 ± 64.73
Triglyceride (mmol/L)	1.52 ± 1.61	1.26 ± 0.59	1.50 ± 0.87
Cholesterol (mmol/L)	4.51 ± 0.85	3.82 ± 1.08*	3.89 ± 0.78
HDL-C (mmol/L)	1.31 ± 0.38	0.89 ± 0.36**	0.68 ± 0.24
LDL-C (mmol/L)	2.64 ± 0.70	2.27 ± 0.89	2.45 ± 0.80
Apolipoprotein A (g/L)	1.27 ± 0.29	0.83 ± 0.29**	0.67 ± 0.20
Creatinine	64.16 ± 14.58	63.78 ± 27.46	67.50 ± 13.48
Urea	6.12 ± 1.21	5.43 ± 2.07	5.95 ± 1.44
Uric acid	378.11 ± 81.82	345.39 ± 143.54	402.00 ± 87.85
eGFR (ml/min/1.73m^2)	111.55 ± 29.90	116.33 ± 37.03	104.28 ± 15.67

WBC, white blood cell counts; RBC, red blood cell counts; HDL-C, high-density lipoprotein cholesterol; LDL-C, low-density lipoprotein cholesterol; ^*^: p < 0.05 vs. Ctrl; ^**^: p < 0.01 vs. Ctrl; ^#^: p < 0.05 vs. ND; ^##^: p < 0.01 vs. ND.

### Sample Preparation and GC/MS Analysis

The plasma was extracted from peripheral blood which was collected using EDTA tubes and then centrifuged at 1000 g for 10 min. The collected plasma samples were immediately frozen at -80°C until metabolomics analysis. All plasma samples were collected from volunteers after overnight fasting (for at least 10 h), and newly diagnosed patients were new diagnosis and prior to any medications.

The metabolomics method based on GC/MS was performed as previously reported (20). Briefly, 50 µL of plasma supernatants was extracted with 200 µL of methanol which contains 5 µg/mL of [^13^C_2_]-myristic acid internal standard (IS). The mixture was vortexed for 5 min and centrifuged (20,000×g, 4°C) for 10 min to remove protein. An aliquot of 100 µL of supernatant was collected and concentrated to dryness. The extract was then oximated with 30 µL of pyridine containing 10 mg/mL methoxyamine for 16 h at room temperature, followed by trimethylsilylation with 30 µL of MSTFA + 1% TMCS for another 1 h. Subsequently, 30 µL of n-heptane containing methyl myristate (15.0 µg/mL) as the quality control reference standard was added and thoroughly mixed. The quality control samples (QC) were pooled with the remaining upper layers of samples in the study set and mixed.

A 0.5 μL portion of the prepared samples was injected into Shimadzu GC/MS QP2010Ultra/SE equipped with a 30 m × 0.25 mm ID, fused silica capillary column, which was chemically bonded with 0.25 m DB1-MS stationary phase. The column temperature was programed as follows: kept at 80°C for 3.0 min, then increased to 300°C at 20°C/min and held for 5.0 min. The transfer line temperature was set at 220°C and the ion source temperature at 200°C. Ions were generated by a 70-eV electron beam at a current of 3.2 mA. The mass spectra were acquired over the mass range of 50-700 m/z at a rate of 25 spectra/s after a solvent delay of 160 s. To diminish the systematic variation during instrumental analysis, all the samples were randomly selected for analysis by GC/MS and QC samples spaced evenly among the injections.

The metabolites were identified by comparing the mass spectrum and retention indexes for the analyte with the corresponding values from Wiley 9, the National Institute of Standards and Technology (NIST) library 2.0, and an in-house mass spectra library database. A match factor greater than 80% is considered to be reliable.

### Statistical Analysis

After identification, statistical analyses were performed using normalized peak area (normalized by IS). The normalized peak area was introduced in SIMCA software for multivariate statistical analysis. The goodness of fit for a model was evaluated using three quantitative parameters as follows: R2X (the explained variation in X), R2Y (the explained variation in Y), and Q2 (the predicted variation in Y based on the model using cross-validation). Statistical analyses were performed using the GraphPad Prism software. Metabolomics pathway analysis of the metabolic biomarkers was carried out using MetaboAnalyst (21). Fold change (FC) was calculated by comparing average level between groups. PET-derived metrics include: tumor maximum standardized uptake value (SUVmax); metabolically active tumor volume (TMTV), calculated as the total volume of tumor with FDG uptake; and total lesion glycolysis (TLG), the sum of the tumor volume weighted by the intensity of FDG uptake. The correlation analysis was performed by R software. Relative distance values (RDV) were calculated as previously reported (22). Survival functions were estimated using the Kaplan-Meier method and compared with the log-rank test. All data are expressed as the means ± SDs. Differences among groups were evaluated by two-tailed Student’s t-test or one-way ANOVA. *P* values less than 0.05 were considered to indicate statistical significance.

## Results

### The Demographics and Clinical Characteristics of DLBCL Patients and Healthy Volunteers

The demographics of all volunteers included in this study are summarized in **Table 1**. All participants were age‐matched and sex‐matched in the Ctrl, ND, and CR groups. Compared with healthy controls, no obvious liver and kidney dysfunction were observed in newly diagnosed DLBCL patients according to the biochemistry analysis. In contrast, abnormal cholesterol metabolism was observed in the ND group as indicated by significantly decreased cholesterol, high-density lipoprotein cholesterol, and apolipoprotein A. The level of lymphocyte, red blood count (RBC), and hemoglobin was also significantly reduced in the ND group, while these laboratory characteristics were restored in the CR group. Moreover, a multivariate statistical model was constructed based on clinical characteristics, showing obvious cluster classification among collected samples (**Supplementary Figure S1A**), and the variable of importance (VIP, **Supplementary Figure S2**) values indicated that lymphocyte, RBC, and hemoglobin contributed the most to distinguishing these three groups. This result suggested that lymphocyte, RBC, and hemoglobin may be the candidate clinical indicators related to diagnosis in DLBCL patients.

### Plasma Metabolic Patterns and Discriminant Metabolites of DLBCL Patients

**Figure 1:**
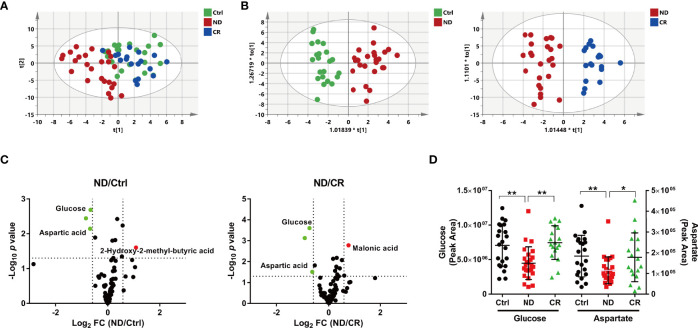
Metabolic patterns and perturbed metabolites of DLBCL patients based on plasma metabolome. **(A)** The PLS-DA score plot, the parameters of the model were: R2X = 0.306, R2Y = 0.351, and Q2 = 0.176. **(B)** The OPLS-DA score plot of the Ctrl group vs. the ND group, the parameters of the model were: R2X = 0.222, R2Y = 0.855, and Q2 = 0.532. The OPLS-DA score plot of the CR group vs. the ND group, the parameters of the model were: R2X = 0.381, R2Y = 0.872, and Q2 = 0.587. **(C)** Volcano maps of the metabolites identified from the Ctrl group and the ND group (left) and the CR group and the ND group (right). Red dots represent metabolites with higher abundance while green dots represent metabolites with lower abundance in the ND group. **(D)** The plasma abundance of glucose and aspartate. ND: patients newly diagnosed; CR: patients achieving complete remission. ^*^: *p*< 0.05; ^**^: *p*< 0.01.

Deconvolution of the plasma chromatograms produced a total of 128 distinct peaks and 87 were authentically identified. The overall process variability was calculated based on the intensity of methyl myristate; the RSD was 6.8%. Moreover, pooled QC samples were clustered well in a PCA plot (**Supplementary Figure S3**), indicating stable instrument operation and good reproducibility of the assay throughout the experiment. To explore the metabolic characteristics among the Ctrl, ND, and CR groups, a supervised PLS-DA model was created to obtain an overview of the data set. The score plot (**Figure 1A**) showed that samples within each group tended to cluster closely, indicating that the metabolic features within each group were similar. Furthermore, the ND group was well separated from the control group, while the CR group moved much closer to the control group, suggesting the metabolic profile could well characterize the disease states of DLBCL. To further quantitatively evaluate the perturbed metabolism, the relative distance values (RDV) were calculated. The RDV between the Ctrl group and the ND group, the CR group was decreased from 7.1 ± 2.1 to 3.3 ± 2.2, indicating the metabolic fluctuation was restored after achieving complete remission. Compared with clinical characteristics (**Supplementary Figures S1B, C**), the OPLS-DA score plots of metabolomics presented better fitness and higher predictability of models for the Ctrl group vs. the ND group (**Figure 1B**) and the CR group vs. the ND group (**Figure 1C**). This indicated the metabolic profiles illustrated stronger recognition among the Ctrl, ND, and CR groups.

To recognize the differential metabolites, volcano plots were processed to display metabolites changed greatly (fold change > 1.5 or < 0.667) and significantly (*p <*0.05). Compared with the control group, 3 metabolites were identified with 2 metabolites decreased (glucose and aspartate) and one metabolite increased (2-hydroxy-2-methyl-butyric acid) in the ND group (**Figure 1C**). While compared with the CR group, 3 metabolites were identified with 2 metabolites decreased (glucose and aspartate) and 1 metabolite increased (malonic acid) in the ND group (**Figure 1C**). These metabolites screened above suggested that glucose and aspartate may be the candidate metabolic biomarkers for the diagnosis in DLBCL patients (**Figure 1D**).

### The Diagnostic Model for DLBCL

**Figure 2:**
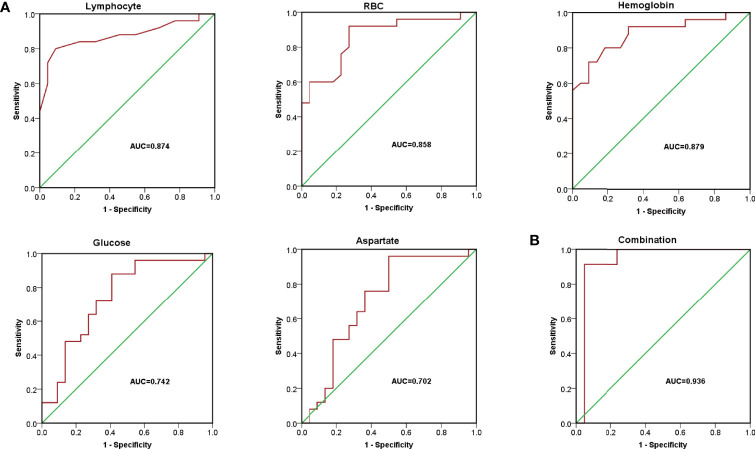
The diagnostic model for DLBCL. **(A)** Receiver operating characteristic (ROC) curve of lymphocyte, RBC, hemoglobin, glucose, and aspartate with AUC= 0.874, 0.858, 0.879, 0.742, and 0.702, respectively. **(B)** ROC curves of the combined equation with AUC = 0.936.

To verify the sensitivity and specificity of the above potential biochemical indicators and metabolic biomarkers for diagnosis, receiver operating characteristic (ROC) curve was performed. As presented in **Figure 2A**, the area under the ROC (ROC-AUC) calculated with lymphocyte, RBC, hemoglobin, glucose, and aspartate achieved a ROC-AUC of 0.874, 0.858, 0.879, 0.742, and 0.702, respectively. To improve the performance of the diagnostic model, combined diagnosis with multiple indexes is usually used. With the metabolic biomarkers (glucose and aspartate) and the hematological indicators (lymphocyte, RBC, and hemoglobin), a logistic regression to establish a diagnostic model was applied to predict diagnosis of DLBCL. The corresponding ROC curve had an AUC of 0.936 (**Figure 2B**) which was higher than that of each candidate biomarker. This indicated that the combined diagnostic model could significantly improve the diagnostic performance with respect to DLBCL.

### Metabolic Biomarkers for Prognosis-Prediction of DLBCL Patients

**Figure 3:**
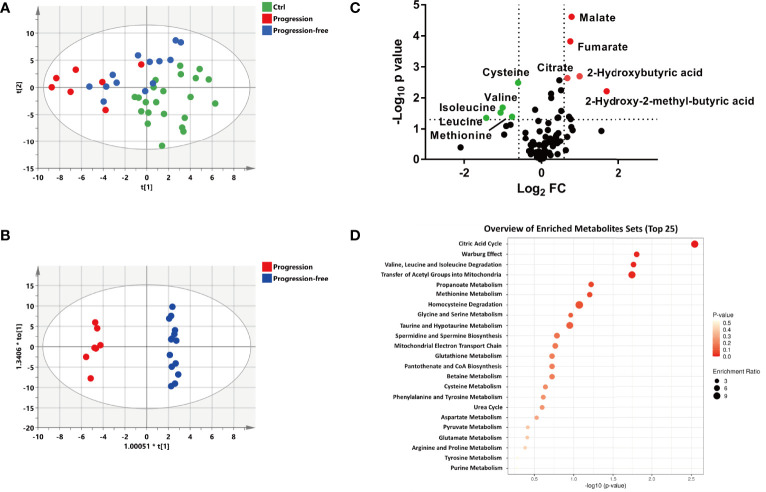
Metabolic patterns and perturbed metabolites associated with the different outcomes of newly diagnosed DLBCL patients. **(A)** The PLS-DA score plot of the Ctrl, progression and progression-free groups, the parameters of the model were: R2X= 0.347, R2Y= 0.468, and Q2 = 0.254. **(B)** The OPLS-DA score plot of the progression and progression-free groups, the parameters of the model were: R2X= 0.594, R2Y= 0.992, and Q2 = 0.558. **(C)** Volcano maps of the metabolites identified from the progression-free group and progression group. Red dots represent metabolites with higher abundance while green dots represent metabolites with lower abundance in the progression group. **(D)** Metabolite pathway enrichment overview of plasma metabolites that displayed statistical significance and changed to a substantial extent.

According to prognostic outcome after follow-up (update to September 2021, four patients were lost to follow-up), the remaining patients in the ND group were divided into the progression-free group (n=14) and the progression group (n=7). A multivariate statistical analysis showed that the progression group was well separated from the control group, while the progression-free group moved much closer to the control group (**Figure 3A**). However, the PLS-DA plots of subgroups divided based on Ann Arbor stage and Han’s algorithm displayed little difference of metabolic profiles (**Supplementary Figures S4A, C**). Furthermore, the OS (overall survival) had no significant difference between stage I-II vs. stage III-IV (*p*=0.998, 95% C.I): 35.5 months (30.0- 41.1 months) vs. 33.4 months (25.4-41.5 month), as well as GCB vs. non-GCB (*p*=0.128, 95% C.I): 38.0 months (32.4-43.6 months) vs. 30.5 months (23.1-37.9 months) (**Supplementary Figures S4B, D**). This suggested that Ann Arbor stage and Han’s algorithm displayed little prediction power for prognosis. The OPLS-DA model showed a clear and distinctive clustering between the samples of the progression-free group and the progression group (**Figure 3B**), indicating that some metabolites contributed to discriminate progression-free patients from progression patients before treatment. This suggested that the plasma metabolic profile of patients before treatment could well predict the prognosis of DLBCL.

**Figure 4:**
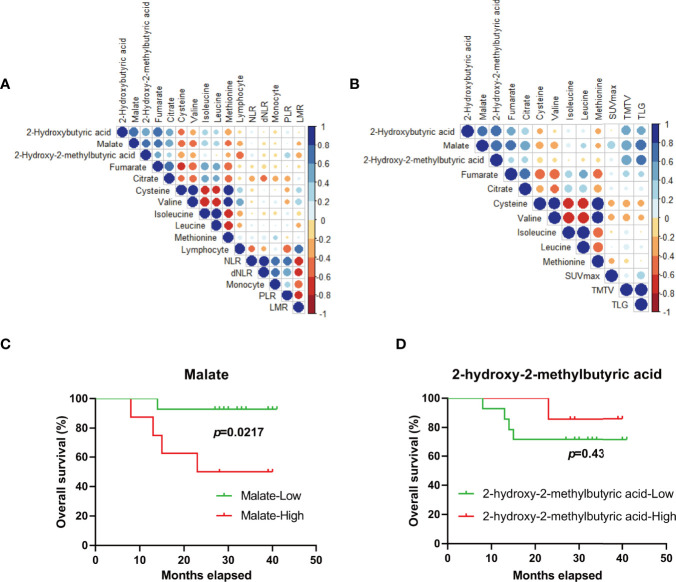
Metabolic biomarkers for outcome-prediction of DLBCL patients. **(A, B)** Pearson’s correlation coefficient between candidate metabolites and potential outcome-predictive indexes including biochemical parameters **(A)** and tumor metrics derived from FDG-PET/CT **(B)** in the clinic. **(C, D)** The survival of DLBCL patients according to malate **(C)** and 2-hydroxy-2-methylbutyric acid **(D)** levels at diagnosis, respectively. Blue color represents a positive correlation while red color represents a negative correlation. NLR, neutrophil/lymphocyte ratio; dNLR, derived neutrophil/lymphocyte ratio; PLR, platelet/lymphocyte ratio; LMR, lymphocyte/monocyte ratio; SUVmax, tumor maximum standardized uptake value; TMTV, metabolically active tumor volume; TLG, total lesion glycolysis.

To identify the biomarkers associated with prognosis, statistically different metabolites (*p* < 0.05) with a large change (fold change> 1.5 or <0.67) between the progression group and the progression-free group were screened out, including 10 metabolites (malate, fumarate, citrate, 2-hydroxy-2-methylbutyric acid, 2-hydroxybutytic acid, cysteine, methionine, leucine, isoleucine, and valine) (**Figure 4C**). The metabolites set enrichment analysis revealed that citric acid cycle, valine, leucine and isoleucine degradation, and methionine metabolism were markedly perturbed (**Figure 4D**).

Some clinic indexes or derived parameters before treatment including neutrophil/lymphocyte ratio (NLR), derived neutrophil/lymphocyte ratio (dNLR), platelet/lymphocyte ratio (PLR), lymphocyte/monocyte ratio (LMR), and monocyte were reported to have the important significance for prognosis-prediction of the patients with DLBCL (23–25). Correlation analysis was performed between the above 8 metabolites and reported indexes (**Figure 4A**), and no significant correlation was found. Apart from staging and response assessment, the baseline ^18^F-fluoro-2-deoxy-D-glucose positron emission tomography (FDG-PET/CT) parameters (SUVmax, TMTV, and TLG) have important prognostic significance for DLBCL patients receiving R-CHOP chemotherapy (26). We were delighted to find that the level of malate and 2-hydroxy-2-methylbutyric acid was positively related with TMTV (r=0.58, *p*=0.015 for malate and r=0.56, *p*=0.018 for 2-hydroxy-2-methylbutyric acid) and TLG (r=0.69, *p*=0.002 for malate and r=0.63, *p*=0.008 for 2-hydroxy-2-methylbutyric acid), respectively (**Figure 4B**). Furthermore, ND patients with higher levels of malate (*p*=0.0217, 25.57 months (15.83-35.31 months) for the high subgroup vs. 39.07 months (35.43-42.71 months) for the low subgroup) not 2-hydroxy-2-methylbutyric acid (*p*=0.43, 37.57 months (33.16- 41.98 months) for the high subgroup vs. 32.85 months (26.07- 39.64 months) for the low subgroup) were associated with a poorer survival (**Figures 4C, D**), which further indicated the malate was a preferential metabolic biomarker for prognosis-prediction of DLBCL patients.

## Discussion

DLBCL is the most common B-cell non-Hodgkin’s lymphoma, while up to 40% of patients become refractory and display a poor survival outcome (27). Efforts to discover biomarkers that uncover coordinate signaling could help to provide a novel perspective of DLBCL. In the present study, we systematically explored the metabolic characteristics of peripheral plasma collected from DLBCL patients and healthy controls, and identified metabolic pathways and biomarkers associated with diagnosis and prognosis-prediction of DLBCL.

### The Differential Metabolic Patterns of DLBCL Patients Displayed Disease Status and Predicted Prognosis

A supervised PLS-DA model revealed that the ND group was well separated from the control group, while the CR group moved much closer to the control group (**Figure 1A**), which was further confirmed by quantitative RDV. It was suggested that the plasma metabolic profile of newly diagnosed DLBCL patients deviated significantly from the healthy controls, and the metabolic fluctuation was restored after achieving complete remission. The OPLS-DA model established with metabolomics data (**Figures 1B, C**) showed better fitness and higher predictability than models with clinical characteristics (**Supplementary Figures S1B, C**), indicating the metabolic profile could well characterize disease states of DLBCL and illustrate stronger recognition among healthy controls, DLBCL patients, and patients after achieving complete remission.

Patients in the ND group were further divided into two groups based on risk stages, subtypes based on Han’s algorithm and prognosis, respectively. The PLS-DA model clearly displayed the poorer prognosis group tended to shift further away from the Ctrl group (**Figure 3A**). However, high-stage subgroups and low-stage subgroups were not well separated, as well as the GCB subgroup and the non-GCB subgroup (**Supplementary Figures S4A, C**) which was consistent with the result reported by Mi et al. (17). This further indicated that information on individual prognosis was contained in the metabolic patterns of pre-treatment bio-fluids. In summary, metabolomics study was informative for us to characterize the metabolic phenotypes, forecast risk and prognosis, and understand the metabolic mechanisms of DLBCL.

### Abnormal Glucose and Aspartate in the Pathogenesis and Diagnosis of DLBCL

The level of glucose and aspartate in plasma was decreased in ND patients and elevated in CR patients, indicating that glucose and aspartate may be involved in the pathogenesis of DLBCL (**Figures 1C, D**). Most tumor cells depend largely on aerobic glycolysis which converts glucose into essential metabolic intermediates for energy production and cell proliferation (28). Pathological lymph nodes of DLBCL also exhibit increasing glucose uptake measured by FDG-PET/CT (12), resulting in a decrease in peripheral glucose. Thus, anti-glycolytic cancer therapy has become a rising research focus for developing anticancer drugs (29), metformin could sensitize therapeutic agents and improve prognosis in pre-clinical and clinical DLBCL (30). More and more evidence demonstrates that aspartate is an endogenous metabolic limitation for tumor growth (31), and up-regulated aspartate metabolism contributed to epithelial-mesenchymal transition in tumor (32). Moreover, the lower level of aspartate in newly diagnosed DLBCL patients may be related to the disorders of asparagine metabolism. Asparaginase catalyzes the hydrolysis of the asparagine into aspartic acid and ammonia and has been used in the treatment of leukemia. Attempts have been made on the effect of asparaginase on DLBCL, and inspiring results were obtained in both the preclinical experiment (33) and clinical trials with a low number of patients (10). More evidence with multi-center clinical trials and an enlarged number of patients are needed to validate the effect of asparaginase on DLBCL.

A clinical diagnosis of DLBCL requires the patient to undergo a pathological biopsy, which is an invasive complex operation (6). Our results suggested that glucose and aspartate were deeply involved in the occurrence and pathogenesis of DLBCL. According to the diagnostic model established based on the identified hematological indicators (**Table 1**) and plasma metabolic indicators (**Figure 1**), favorable performance (AUC =0.936) was achieved with the combination of five candidate indicators, which was higher than with each single indicator (**Figure 2B**).

### Disrupted Metabolic Pathways Related to Prognosis and Underlying Mechanism

**Figure 5:**
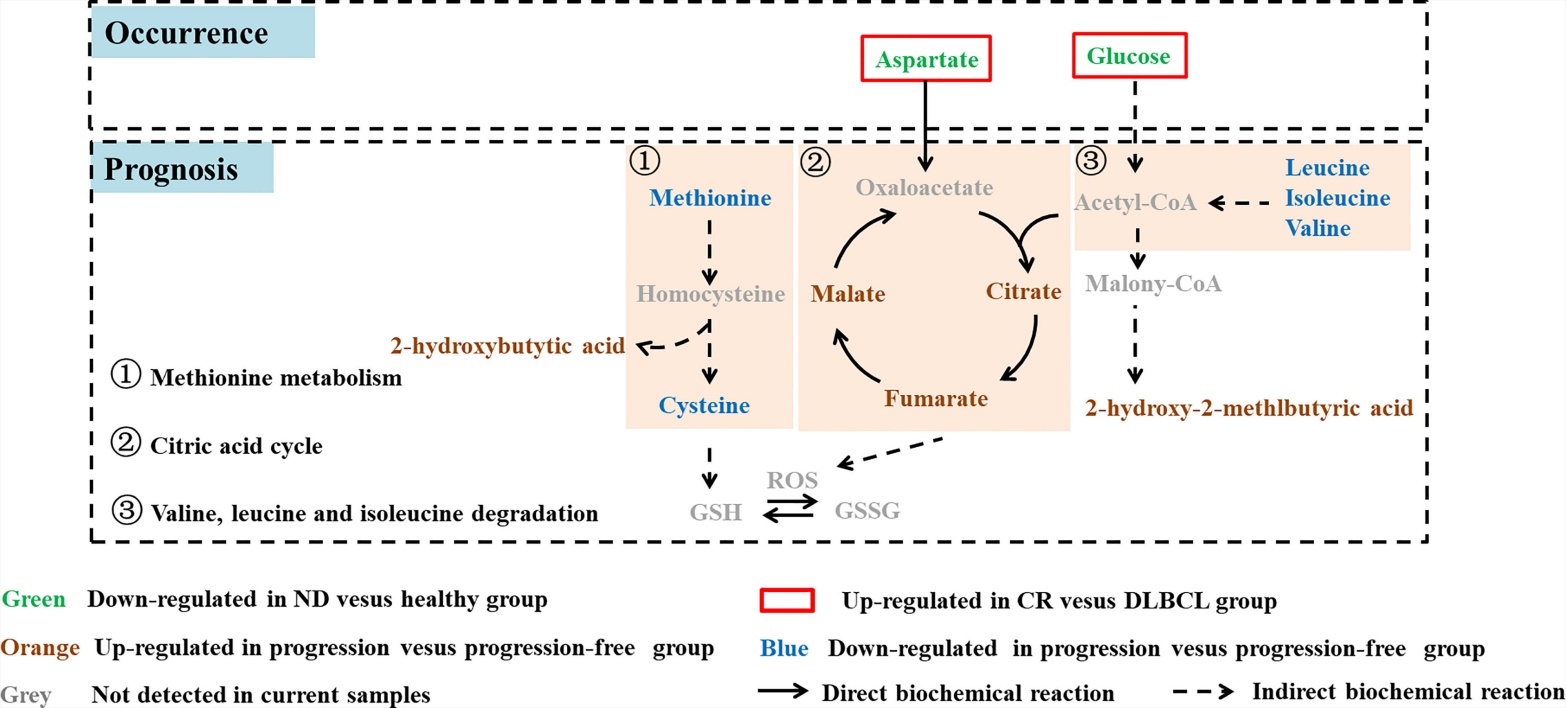
Typical metabolites and associated pathways perturbed in DLBCL patients.

According to the prognostic outcome, we found that patients with poor prognosis showed vigorous oxidative phosphorylation (OxPhos) metabolism indicated by enrichment of typical metabolites in a citric acid cycle (TCA), such as citrate, fumarate, and malate (**Figures 3** and **5**). It has been reported that the subtype of DLBCL cell lines which rely on mitochondrial energy transduction called OxPhos-DLBCLs provide pro-survival benefits (34) and the intermediates in the TCA cycle have a multifaceted contribution to tumor progression (35). Citrate is a critical metabolic checkpoint involved in several important metabolic pathways, a certain level of citrate is necessary for cancer cell proliferation and tumor growth by the production of acetyl-CoA which is important for lipogenesis, membrane expansion, and acetylation (36). Fumarate is another physiological metabolic intermediate of the TCA cycle, the accumulation of fumarate which could permeate multiple subcellular compartments (37) as well as an extracellular microenvironment (38). The abnormally increased fumarate is a key activator of a variety of oncogenic cascades (39). Growing evidence supports the idea that malate accumulation may be essential for cancer growth by contributing to both redox balance (40, 41) and an elevated glycolytic flux (42, 43). Hence, it has seemed that the flux of oxidative phosphorylation was closely related to tumor growth and progression, which can indicate the prognosis of the tumor.

In addition, an obvious decline of methionine and cysteine and consistent decrease of branched-chain amino acids (BCAAs) were observed in the plasma of DLBCL patients with poor prognosis. Valine, leucine, and isoleucine are essential BCAAs whose metabolism can both influence multiple cancer phenotypes and serve as a marker of disease pathology (44). Valine was reported as a candidate biomarker for the prognosis of DLBCL by Stenson et al. (18) and Mi et al. (17). It is likely that the degradation of BCAAs to acetyl-CoA is enhanced, which further supports the generation of TCA cycle intermediates (**Figure 5**) to fuel cancer growth. Methionine metabolism coordinates nucleotide and redox status which are relevant to cancer pathogenesis (45). The decreased methionine and cysteine may maintain cellular redox status by producing the antioxidant glutathione (GSH) against reactive oxygen species (ROS) which was generated in remarkable OxPhos metabolism (**Figure 5**). Elevated 2-hydroxybutyric acid, a byproduct of glutathione anabolism, indirectly indicated the robust requirement to maintain the redox state (**Figure 5**). Moreover, 2-hydroxybutyric acid has previously been identified as a biomarker of diabetes risk (46, 47) and mitochondrial disorders (48). This suggested that DLBCL patients with higher risk of diabetes were more likely to have a poor prognosis. 2-hydroxy-2-methlbutyric acid belongs to hydroxy fatty acid and little is known about its biological functions. Further study is needed to exploit its role in DLBCL. Overall, elucidation of the metabolic mechanisms underlying the DLBCL progression is still needed in our further study.

### Malate as the Potential Plasma Metabolic Biomarker for Prognosis-Prediction of DLBCL

Due to the heterogeneity and different prognosis of DLBCL patients, it is urgent to exploit new biomarkers for prognosis-prediction. Candidate metabolites including malate, fumarate, citrate, 2-hydroxy-2-methylbutyric acid, cysteine, methionine, leucine, isoleucine, and valine were first screened out, correlation analysis was then performed to evaluate the talent of these markers. No significant correlation was found between the above metabolites and reported biochemical parameters which have predictive power to predict prognosis (**Figure 4A**). It is probably because these reported parameters (derived from lymphocytes, neutrophils, and monocyte) just reflect the abnormal immune responses which is an essential pathogenic factor for DLBCL (49), while metabolic markers, signs of metabolic status, may be independent risk factors and could not be simply associated with hematological parameters.

Functional imaging with FDG integrated with PET-CT provides valuable measurements of tumor metabolism and activity of DLBCL. The derived tumor metrics (SUVmax, TMTV, and TLG) not only has been affirmed in metabolic characteristics, staging, and end-of-treatment evaluation, but they have the potential to improve the accuracy of prognostication (50). Interestingly, the level of malate and 2-hydroxy-2-methylbutyric acid was positively related with TMTV and TLG (**Figure 4B**). Furthermore, ND patients with higher levels of malate but not 2-hydroxy-2-methylbutyric acid had a poorer survival (**Figures 4C, D**). Thus, malate was a preferential plasma metabolic biomarker for prognosis-prediction of DLBCL patients. Attempts should be made to get more samples to further validate our result on a larger scale, and the role of malate metabolism in DLBCL will be verified in subsequent studies.

There were still some limitations in this study. Although the interference of metabolic diseases such as diabetes was excluded as much as possible, the influence of other concomitant diseases was still unknown. Furthermore, a validation dataset with more samples is needed, which is underway by us to verify these results.

## Conclusion

In this study, distinct metabolic features of DLBCL patients in different disease status (before treatment and achieving complete remission) was displayed, and metabolic profiles of newly diagnosed DLBCL patients undergoing different prognosis showed clear and distinctive clustering indicating prognosis-prediction capacity. Based on screened metabolites and clinical indicators, a distinct diagnostic equation was established and improved the diagnostic performance. We found specific disturbance of TCA metabolism, BCAA metabolism, and methionine metabolism in newly diagnosed DLBCL patients was strongly associated with poor prognosis. In addition, malate was further identified as a potentially unfavorable metabolic biomarker for prognosis-prediction of DLBCL. However, the hidden mechanism of abnormal key metabolic pathways especially malate on pathogenesis of DLBCL should be explored in future work.

## Data Availability Statement

The raw data supporting the conclusions of this article will be made available by the authors, without undue reservation.

## Ethics Statement

The studies involving human participants were reviewed and approved by The Medical Ethical Committee of Nanjing Drum Tower Hospital. The patients/participants provided their written informed consent to participate in this study.

## Author Contributions

FF performed the untargeted metabolomics, conducted the data analysis, and wrote the manuscript. MHZ and ZZX collected plasma samples and information of medical history and prognosis. RBS helped with reviewing the manuscript. JL, BC, and XC were lead principal investigators, designed the experiments, supervised progress, and approved the final version of the article. All authors contributed to the article and approved the submitted version.

## Funding

This study was financially supported by the National Natural Science Foundation of China (31371399, 81503139) and the Natural Science Foundation of Nanjing University of Chinese Medicine (XZR2020055).

## Conflict of Interest

The authors declare that the research was conducted in the absence of any commercial or financial relationships that could be construed as a potential conflict of interest.

## Publisher’s Note

All claims expressed in this article are solely those of the authors and do not necessarily represent those of their affiliated organizations, or those of the publisher, the editors and the reviewers. Any product that may be evaluated in this article, or claim that may be made by its manufacturer, is not guaranteed or endorsed by the publisher.

## References

[1] Swerdlow SH Campo E Pileri SA Harris NL Stein H Siebert R. The 2016 Revision of the World Health Organization Classification of Lymphoid Neoplasms. Blood (2016) 127(20):2375–90. doi: 10.1182/blood-2016-01-643569 PMC4874220 26980727

[2] Sehn LH Gascoyne RD. Diffuse Large B-Cell Lymphoma: Optimizing Outcome in the Context of Clinical and Biologic Heterogeneity. Blood (2015) 125(1):22–32. doi: 10.1182/blood-2014-05-577189 25499448

[3] International Non-Hodgkin's Lymphoma Prognostic Factors P. A Predictive Model for Aggressive Non-Hodgkin's Lymphoma. N Engl J Med (1993) 329(14):987–94. doi: 10.1056/NEJM199309303291402 8141877

[4] Ninan MJ Wadhwa PD Gupta P. Prognostication of Diffuse Large B-Cell Lymphoma in the Rituximab Era. Leuk Lymph (2011) 52(3):360–73. doi: 10.3109/10428194.2010.543716 21275631

[5] Alizadeh AA Eisen MB Davis RE Ma C Lossos IS Rosenwald A. Distinct Types of Diffuse Large B-Cell Lymphoma Identified by Gene Expression Profiling. Nature (2000) 403(6769):503–11. doi: 10.1038/35000501 10676951

[6] Liu Y Barta SK. Diffuse Large B-Cell Lymphoma: 2019 Update on Diagnosis, Risk Stratification, and Treatment. Am J Hematol (2019) 94(5):604–16. doi: 10.1002/ajh.25460 30859597

[7] Gu K Weisenburger DD Fu K Chan WC Greiner TC Aoun P. Cell of Origin Fails to Predict Survival in Patients With Diffuse Large B-Cell Lymphoma Treated With Autologous Hematopoietic Stem Cell Transplantation. Hematol Oncol (2012) 30(3):143–9. doi: 10.1002/hon.1017 PMC4524810 22009820

[8] Moskowitz CH Zelenetz AD Kewalramani T Hamlin P Lessac-Chenen S Houldsworth J. Cell of Origin, Germinal Center Versus Nongerminal Center, Determined by Immunohistochemistry on Tissue Microarray, Does Not Correlate With Outcome in Patients With Relapsed and Refractory DLBCL. Blood (2005) 106(10):3383–5. doi: 10.1182/blood-2005-04-1603 16091454

[9] Costa LJ Maddocks K Epperla N Reddy NM Karmali R Umyarova E. Diffuse Large B-Cell Lymphoma With Primary Treatment Failure: Ultra-High Risk Features and Benchmarking for Experimental Therapies. Am J hematol (2017) 92(2):161–70. doi: 10.1002/ajh.24615 PMC5549936 27880984

[10] Chiche J Reverso-Meinietti J Mouchotte A Rubio-Patino C Mhaidly R Villa E. GAPDH Expression Predicts the Response to R-CHOP, the Tumor Metabolic Status, and the Response of DLBCL Patients to Metabolic Inhibitors. Cell Metab (2019) 29(6):1243–57.e10. doi: 10.1016/j.cmet.2019.02.002 30827861

[11] Vander Heiden MG DeBerardinis RJ. Understanding the Intersections Between Metabolism and Cancer Biology. Cell (2017) 168(4):657–69. doi: 10.1016/j.cell.2016.12.039 PMC5329766 28187287

[12] Shim HK Lee WW Park SY Kim H So Y Kim SE. Expressions of Glucose Transporter Types 1 and 3 and Hexokinase-II in Diffuse Large B-Cell Lymphoma and Other B-Cell non-Hodgkin's Lymphomas. Nucl Med Biol (2009) 36(2):191–7. doi: 10.1016/j.nucmedbio.2008.11.009 19217531

[13] Bhalla K Jaber S Nahid MN Underwood K Beheshti A Landon A. Role of Hypoxia in Diffuse Large B-Cell Lymphoma: Metabolic Repression and Selective Translation of HK2 Facilitates Development of DLBCL. Sci Rep (2018) 8(1):744. doi: 10.1038/s41598-018-19182-8 29335581 PMC5768748

[14] Kim J DeBerardinis RJ. Mechanisms and Implications of Metabolic Heterogeneity in Cancer. Cell Metab (2019) 30(3):434–46. doi: 10.1016/j.cmet.2019.08.013 PMC6730674 31484055

[15] Tong Y Gao WQ Liu Y. Metabolic Heterogeneity in Cancer: An Overview and Therapeutic Implications. Biochim Biophys Acta Rev cancer (2020) 1874(2):188421. doi: 10.1016/j.bbcan.2020.188421 32835766

[16] Monti S Savage KJ Kutok JL Feuerhake F Kurtin P Mihm M. Molecular Profiling of Diffuse Large B-Cell Lymphoma Identifies Robust Subtypes Including One Characterized by Host Inflammatory Response. Blood (2005) 105(5):1851–61. doi: 10.1182/blood-2004-07-2947 15550490

[17] Mi M Liu Z Zheng X Wen Q Zhu F Li J. Serum Metabolomic Profiling Based on GC/MS Helped to Discriminate Diffuse Large B-Cell Lymphoma Patients With Different Prognosis. Leuk Res (2021) 111:106693. doi: 10.1016/j.leukres.2021.106693 34455197

[18] Stenson M Pedersen A Hasselblom S Nilsson-Ehle H Karlsson BG Pinto R. Serum Nuclear Magnetic Resonance-Based Metabolomics and Outcome in Diffuse Large B-Cell Lymphoma Patients - A Pilot Study. Leuk lymph (2016) 57(8):1814–22. doi: 10.3109/10428194.2016.1140164 26887805

[19] Hans CP Weisenburger DD Greiner TC Gascoyne RD Delabie J Ott G. Confirmation of the Molecular Classification of Diffuse Large B-Cell Lymphoma by Immunohistochemistry Using a Tissue Microarray. Blood (2004) 103(1):275–82. doi: 10.1182/blood-2003-05-1545 14504078

[20] Aa JY Wang GJ Hao HP Huang Q Lu YH Yan B. Differential Regulations of Blood Pressure and Perturbed Metabolism by Total Ginsenosides and Conventional Antihypertensive Agents in Spontaneously Hypertensive Rats. Acta pharmacol Sinica (2010) 31(8):930–7. doi: 10.1038/aps.2010.86 PMC4091415 20686518

[21] Xia JG Sinelnikov IV Han B Wishart DS. MetaboAnalyst 3.0-Making Metabolomics More Meaningful. Nucleic Acids Res (2015) 43(W1):W251–7. doi: 10.1093/nar/gkv380 PMC4489235 25897128

[22] Aa JY Shao F Wang GJ Huang Q Zha WB Yan B. Gas Chromatography Time-of-Flight Mass Spectrometry Based Metabolomic Approach to Evaluating Toxicity of Triptolide. Metabolomics (2011) 7(2):217–25. doi: 10.1007/s11306-010-0241-8

[23] Wang S Ma Y Sun L Shi Y Jiang S Yu K. Prognostic Significance of Pretreatment Neutrophil/Lymphocyte Ratio and Platelet/Lymphocyte Ratio in Patients With Diffuse Large B-Cell Lymphoma. BioMed Res Int (2018) 2018:9651254. doi: 10.1155/2018/9651254 30643825 PMC6311253

[24] Wilcox RA Ristow K Habermann TM Inwards DJ Micallef IN Johnston PB. The Absolute Monocyte and Lymphocyte Prognostic Score Predicts Survival and Identifies High-Risk Patients in Diffuse Large-B-Cell Lymphoma. Leukemia (2011) 25(9):1502–9. doi: 10.1038/leu.2011.112 21606957

[25] Matsuki E Bohn OL El Jamal S Pichardo JD Zelenetz AD Younes A. Lymphocyte-To-Monocyte Ratio May Serve as a Better Prognostic Indicator Than Tumor-Associated Macrophages in DLBCL Treated With Rituximab. Appl immunohistochem Mol morphol AIMM (2019) 27(8):572–80. doi: 10.1097/PAI.0000000000000645 PMC6374215 30106758

[26] Islam P Goldstein J Flowers CR. PET-Derived Tumor Metrics Predict DLBCL Response and Progression-Free Survival. Leuk Lymph (2019) 60(8):1965–71. doi: 10.1080/10428194.2018.1562181 PMC6635064 30714446

[27] Wang L Li LR Young KH. New Agents and Regimens for Diffuse Large B Cell Lymphoma. J Hematol Oncol (2020) 13(1):175. doi: 10.1186/s13045-020-01011-z 33317571 PMC7734862

[28] Lunt SY Vander Heiden MG. Aerobic Glycolysis: Meeting the Metabolic Requirements of Cell Proliferation. Annu Rev Cell Dev Biol (2011) 27:441–64. doi: 10.1146/annurev-cellbio-092910-154237 21985671

[29] Abdel-Wahab AF Mahmoud W Al-Harizy RM. Targeting Glucose Metabolism to Suppress Cancer Progression: Prospective of Anti-Glycolytic Cancer Therapy. Pharmacol Res (2019) 150:104511. doi: 10.1016/j.phrs.2019.104511 31678210

[30] Singh AR Gu JJ Zhang Q Torka P Sundaram S Mavis C. Metformin Sensitizes Therapeutic Agents and Improves Outcome in Pre-Clinical and Clinical Diffuse Large B-Cell Lymphoma. Canc Metab (2020) 8:10. doi: 10.1186/s40170-020-00213-w PMC7336499 32647571

[31] Sullivan LB Luengo A Danai LV Bush LN Diehl FF Hosios AM. Aspartate is an Endogenous Metabolic Limitation for Tumour Growth. Nat Cell Biol (2018) 20(7):782–8. doi: 10.1038/s41556-018-0125-0 PMC6051729 29941931

[32] Chen Y Wang K Liu T Chen J Lv W Yang W. Decreased Glucose Bioavailability and Elevated Aspartate Metabolism in Prostate Cancer Cells Undergoing Epithelial-Mesenchymal Transition. J Cell Physiol (2020) 235(7-8):5602–12. doi: 10.1002/jcp.29490 32017073

[33] Eraslan Z Papatzikas G Cazier JB Khanim FL Gunther UL. Targeting Asparagine and Serine Metabolism in Germinal Centre-Derived B Cells Non-Hodgkin Lymphomas (B-NHL). Cells (2021) 10(10):2589. doi: 10.3390/cells10102589 34685569 PMC8533740

[34] Norberg E Lako A Chen PH Stanley IA Zhou F Ficarro SB. Differential Contribution of the Mitochondrial Translation Pathway to the Survival of Diffuse Large B-Cell Lymphoma Subsets. Cell Death Differ (2017) 24(2):251–62. doi: 10.1038/cdd.2016.116 PMC5299709 27768122

[35] Eniafe J Jiang S. The Functional Roles of TCA Cycle Metabolites in Cancer. Oncogene (2021) 40(19):3351–63. doi: 10.1038/s41388-020-01639-8 33864000

[36] Huang L Wang C Xu H Peng G. Targeting Citrate as a Novel Therapeutic Strategy in Cancer Treatment. Biochim Biophys Acta Rev Canc (2020) 1873(1):188332. doi: 10.1016/j.bbcan.2019.188332 31751601

[37] O'Flaherty L Adam J Heather LC Zhdanov AV Chung YL Miranda MX. Dysregulation of Hypoxia Pathways in Fumarate Hydratase-Deficient Cells is Independent of Defective Mitochondrial Metabolism. Hum Mol Genet (2010) 19(19):3844–51. doi: 10.1093/hmg/ddq305 PMC2935862 20660115

[38] Goncalves E Sciacovelli M Costa ASH Tran MGB Johnson TI Machado D. Post-Translational Regulation of Metabolism in Fumarate Hydratase Deficient Cancer Cells. Metab engineer (2018) 45:149–57. doi: 10.1016/j.ymben.2017.11.011 PMC5805855 29191787

[39] Schmidt C Sciacovelli M Frezza C. Fumarate Hydratase in Cancer: A Multifaceted Tumour Suppressor. Semin Cell Dev Biol (2020) 98:15–25. doi: 10.1016/j.semcdb.2019.05.002 31085323 PMC6974395

[40] Son J Lyssiotis CA Ying H Wang X Hua S Ligorio M. Glutamine Supports Pancreatic Cancer Growth Through a KRAS-Regulated Metabolic Pathway. Nature (2013) 496(7443):101–5. doi: 10.1038/nature12040 PMC3656466 23535601

[41] Lim JKM Leprivier G. The Impact of Oncogenic RAS on Redox Balance and Implications for Cancer Development. Cell Death Dis (2019) 10(12):955. doi: 10.1038/s41419-019-2192-y 31852884 PMC6920345

[42] Zhang B Tornmalm J Widengren J Vakifahmetoglu-Norberg H Norberg E. Characterization of the Role of the Malate Dehydrogenases to Lung Tumor Cell Survival. J Canc (2017) 8(11):2088–96. doi: 10.7150/jca.19373 PMC5559971 28819410

[43] Hanse EA Ruan C Kachman M Wang D Lowman XH Kelekar A. Cytosolic Malate Dehydrogenase Activity Helps Support Glycolysis in Actively Proliferating Cells and Cancer. Oncog (2017) 36(27):3915–24. doi: 10.1038/onc.2017.36 PMC5501748 28263970

[44] Sivanand S Vander Heiden MG. Emerging Roles for Branched-Chain Amino Acid Metabolism in Cancer. Canc Cell (2020) 37(2):147–56. doi: 10.1016/j.ccell.2019.12.011 PMC7082774 32049045

[45] Sanderson SM Gao X Dai Z Locasale JW. Methionine Metabolism in Health and Cancer: A Nexus of Diet and Precision Medicine. Nat Rev Canc (2019) 19(11):625–37. doi: 10.1038/s41568-019-0187-8 31515518

[46] Gall WE Beebe K Lawton KA Adam KP Mitchell MW Nakhle PJ. Alpha-Hydroxybutyrate Is an Early Biomarker of Insulin Resistance and Glucose Intolerance in a Nondiabetic Population. PloS One (2010) 5(5):e10883. doi: 10.1371/journal.pone.0010883 20526369 PMC2878333

[47] Ferrannini E Natali A Camastra S Nannipieri M Mari A Adam KP. Early Metabolic Markers of the Development of Dysglycemia and Type 2 Diabetes and Their Physiological Significance. Diabetes (2013) 62(5):1730–7. doi: 10.2337/db12-0707 PMC3636608 23160532

[48] Thompson Legault J Strittmatter L Tardif J Sharma R Tremblay-Vaillancourt V Aubut C. A Metabolic Signature of Mitochondrial Dysfunction Revealed Through a Monogenic Form of Leigh Syndrome. Cell Rep (2015) 13(5):981–9. doi: 10.1016/j.celrep.2015.09.054 PMC4644511 26565911

[49] Pasqualucci L Dalla-Favera R. Genetics of Diffuse Large B-Cell Lymphoma. Blood (2018) 131(21):2307–19. doi: 10.1182/blood-2017-11-764332 PMC5969374 29666115

[50] Thanarajasingam G Bennani-Baiti N Thompson CA. PET-CT in Staging, Response Evaluation, and Surveillance of Lymphoma. Curr Treat options Oncol (2016) 17(5):24. doi: 10.1007/s11864-016-0399-z 27032646

