# Supplementary material for: Plasma Metabolites Forecast Occurrence and Prognosis for Patients With Diffuse Large B-Cell Lymphoma
Source: Front Oncol. 2022 Jun 6;12:894891. doi: 10.3389/fonc.2022.894891 (PMC9207198; doi:10.3389/fonc.2022.894891)
Supplement: Supplementary file 1 [file DataSheet_1.docx]

**Supporting information**

**Plasma metabolites forecast occurrence and prognosis for patients with diffuse large B-cell lymphoma**

Fei Fei^1^, Meihong Zheng^1^, Zhenzhen Xu^1^, Runbin Sun^1^, Xin Chen^2, *^, Bei Cao^1, *^, Juan Li^1, *^

^1^ Phase I Clinical Trials Unit, The Affiliated Drum Tower Hospital of Nanjing University Medical School, Nanjing, China

^2^ Department of General Surgery, The Affiliated Drum Tower Hospital of Nanjing University Medical School, Nanjing, China

*: Corresponding author:

Xin Chen [xinchen_njglyy@126.com](mailto:xinchen_njglyy@126.com)

Bei Cao [cb_cpu@163.com](mailto:cb_cpu@163.com)

Juan Li [juanli2003@163.com](mailto:juanli2003@163.com)

**Page S-3: Figure S1** Multivariate statistical analysis of clinical characteristics in DLBCL patients and healthy volunteers. (A) The PLS-DA score plot, the parameters of the model were: R2X= 0.308, R2Y= 0.35, Q2= 0.151. (B) The OPLS-DA score plot of the Ctrl group vs. ND group, the parameters of the model were: R2X= 0.333, R2Y= 0.599, Q2= 0. 435. (C) The OPLS-DA score plot of the CR group vs. the ND group, the parameters of the model were: R2X= 0.296, R2Y =0.557, Q2= 0.404. ND: patients newly diagnosed; CR: patients achieving complete remission.

**Page S-4: Figure S2** The VIP values of clinical characteristics. WBC: White blood cell counts; RBC: Red blood cell counts; HDL-C: High-density lipoprotein cholesterol; LDL-C: Low-density lipoprotein cholesterol.

**Page S-5: Figure S3** Metabolic patterns (PCA plot) of DLBCL patients and healthy controls based on plasma metabolome, the parameters of the model were: R2X = 0.357, Q2 = 0.245. ND: patients newly diagnosed; CR: patients achieving complete remission.

**Page S-6: Figure S4** Metabolic shifts and survival of DLBCL subgroups. (A) The PLS-DA score plot of DLBCL subgroups with different stages. (B) The survival of DLBCL patients in different stages. (C) The PLS-DA score plot of DLBCL subgroups with different subtypes based on the cell-of-origin classification. (D) The survival of DLBCL patients in different subtypes. ND-Ⅰ/Ⅱ/Ⅲ/Ⅳ: International staging system stage Ⅰ/Ⅱ/Ⅲ/Ⅳ; GCB: the germinal center B cell subtype.


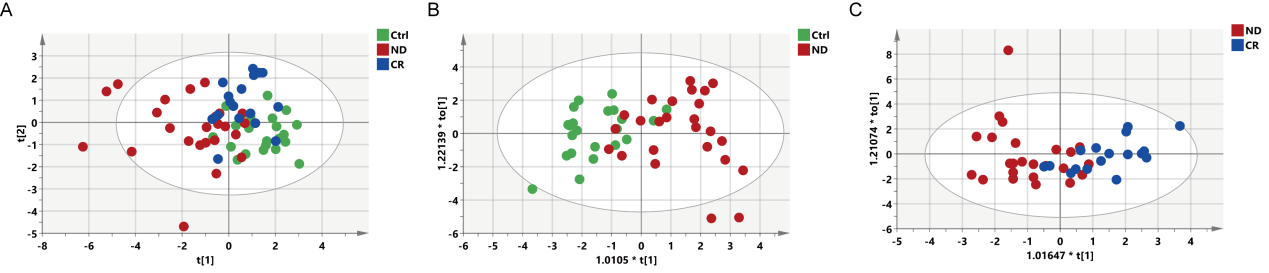


Figure S1 Multivariate statistical analysis of clinical characteristics in DLBCL patients and healthy volunteers. (A) The PLS-DA score plot, the parameters of the model were: R2X= 0.308, R2Y= 0.35, Q2= 0.151. (B) The OPLS-DA score plot of the Ctrl group vs. ND group, the parameters of the model were: R2X= 0.333, R2Y= 0.599, Q2= 0. 435. (C) The OPLS-DA score plot of the CR group vs. the ND group, the parameters of the model were: R2X= 0.296, R2Y =0.557, Q2= 0.404. ND: patients newly diagnosed; CR: patients achieving complete remission.


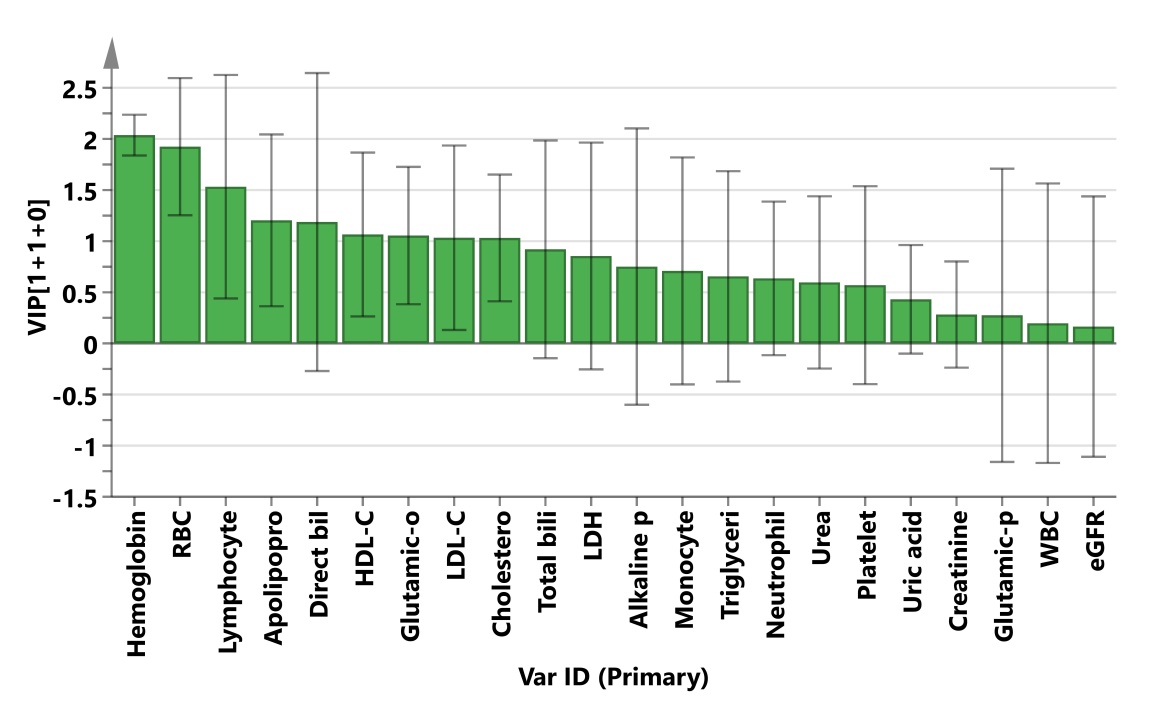


Figure S2 The VIP values of clinical characteristics. WBC: White blood cell counts; RBC: Red blood cell counts; HDL-C: High-density lipoprotein cholesterol; LDL-C: Low-density lipoprotein cholesterol.


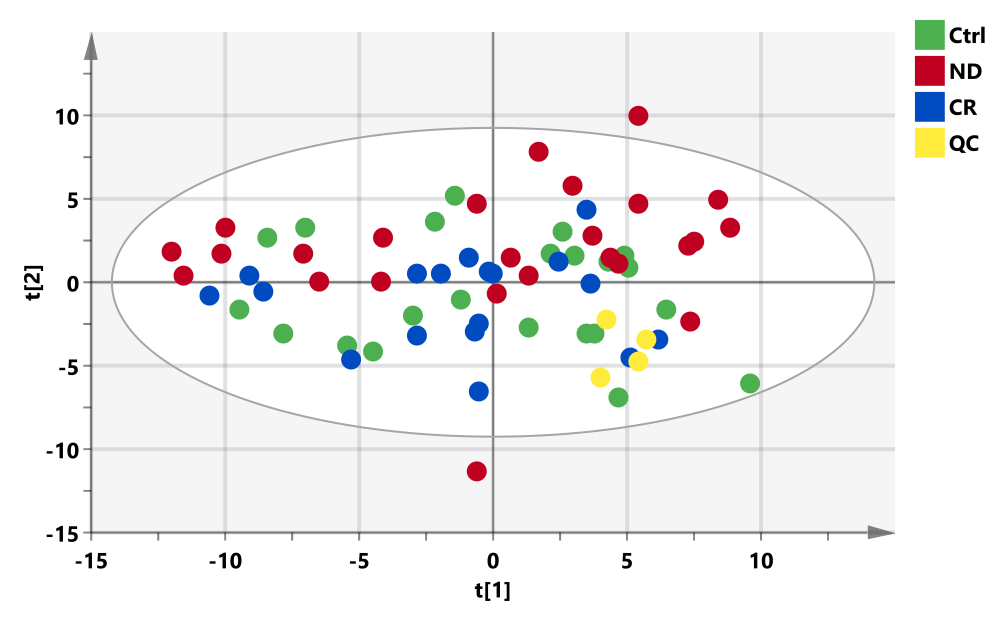


Figure S3 Metabolic patterns (PCA plot) of DLBCL patients and healthy controls based on plasma metabolome, the parameters of the model were: R2X = 0.357, Q2 = 0.245. ND: patients newly diagnosed; CR: patients achieving complete remission.


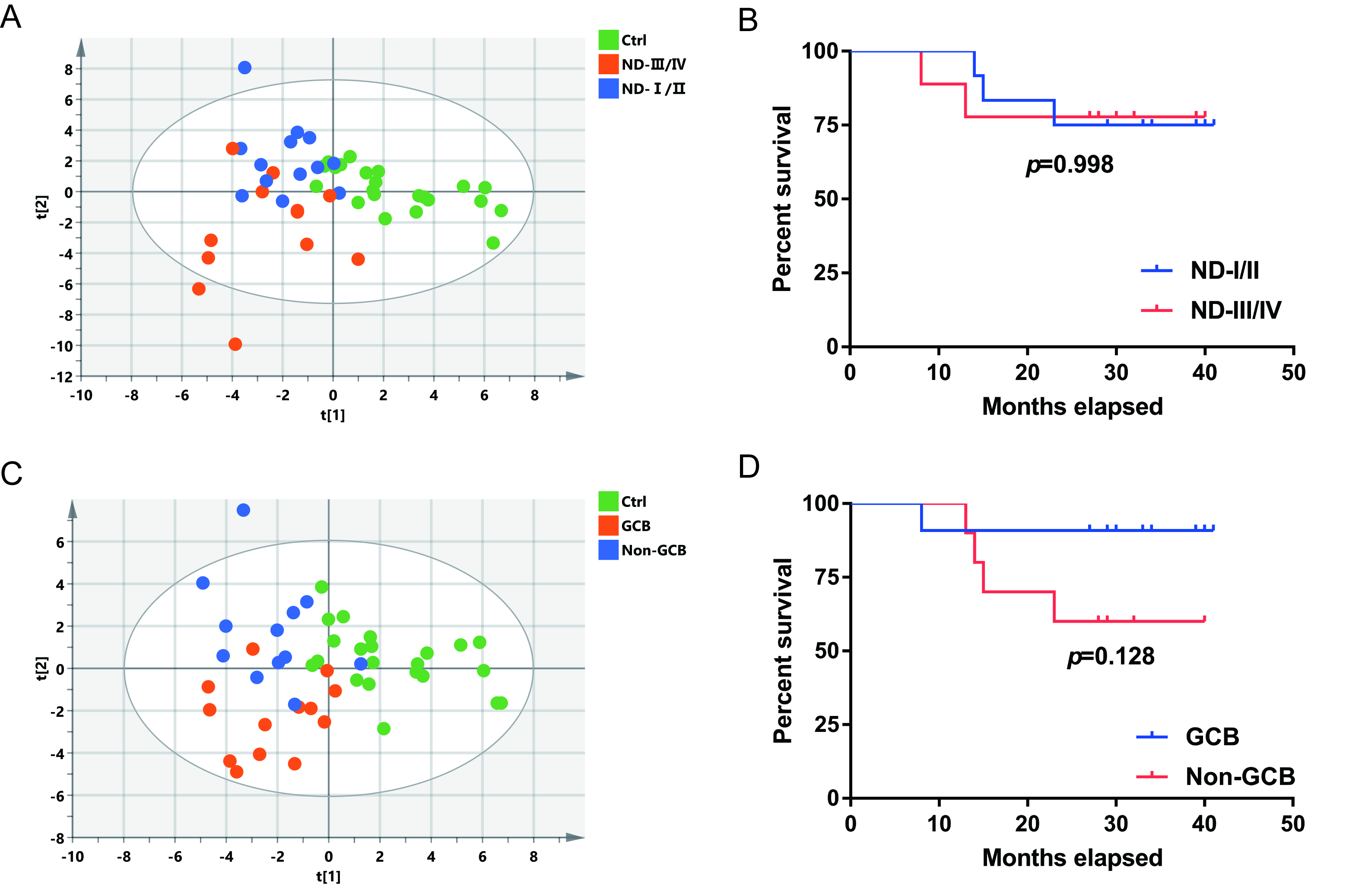


Figure S4 Metabolic shifts and survival of DLBCL subgroups. (A) The PLS-DA score plot of DLBCL subgroups with different stages. (B) The survival of DLBCL patients in different stages. (C) The PLS-DA score plot of DLBCL subgroups with different subtypes based on the cell-of-origin classification. (D) The survival of DLBCL patients in different subtypes. ND-Ⅰ/Ⅱ/Ⅲ/Ⅳ: International staging system stage Ⅰ/Ⅱ/Ⅲ/Ⅳ; GCB: the germinal center B cell subtype.
